# Atrioventricular block can be used as a risk predictor of clinical atrial fibrillation

**DOI:** 10.1002/clc.23167

**Published:** 2019-03-18

**Authors:** Xiao Zhao, Chaofeng Sun, Miaomiao Cao, Hao Li

**Affiliations:** ^1^ Health Science Center Xi'an Jiaotong University Xi'an P.R.China; ^2^ Cardiovascular Department, The First Affiliated Hospital Xi'an Jiaotong University Xi'an P.R.China; ^3^ Department of Rehabilitation and Treatment, the First Affiliated Hospital Xi'an Jiaotong University Xi'an P.R.China

**Keywords:** I AVB, II AVB, III AVB, atrial fibrillation, risk indicator

## Abstract

**Background:**

Atrial fibrillation (AF) is the most common cardiac arrhythmia, with its incidence making up nearly one‐third of all hospital admissions. Atrioventricular block (AVB) is a conduction abnormality along the atrioventricular node or the His‐Purkinje system. The relationship between atrioventricular conduction block and AF is controversial.

**Hypothesis:**

This study is designed to observe whether there is a correlation between AVB and AF, and which type of AVB has the most obvious correlation with AF.

**Methods:**

This study retrospectively reviewed 1345 patients. We classified the AVB according to the AVB classification criteria. One hundred and two patients were excluded, and the final total sample size was 1243 patients, including 679 patients in the AF group (378, 55.7% males) and 564 patients in the non‐AF group (287, 50.8% males). AF group and non‐AF group were compared to observe the relationship between AVB and AF.

**Results:**

The **I** AVB have a relative statistical risk of 1.927 (95% confidence interval [CI]: 1.160‐3.203, *P* < 0.05) with the occurrence of AF. II AVB occupied the largest proportion, accounting for 67 cases (9.87%), and the statistical risk of II AVB in AF is 16.845 (95% CI: 6.099‐46.524, *P* < 0.000). III AVB has a comparative statistical risk of 17.599 (95% CI: 4.212‐73.541, *P* < 0.000).

**Conclusions:**

The three types of AVB in the AF group were significantly higher than that in the non‐AF group. II AVB has the highest incidence rate compared with other types of AVB in the AF group. AVB can be used as a risk factor for AF occurrence.

## 1 INTRODUCTION

Atrial fibrillation (AF) is the most common sustained cardiac arrhythmia in clinical practice, and its incidence is increasing rapidly worldwide, accounting for nearly one‐third of all hospital admissions.^1^ Meanwhile, it is also associated with increased risks of stroke, heart failure, cognitive dysfunction, impaired quality of life, and substantial healthcare costs. Furthermore, it eventually contributes to the increased risks of thromboembolic events,^2^ cardiac, and overall mortalities.^3^, ^4^, ^5^, ^6^ Atrioventricular block (AVB) is a conduction abnormality along the atrioventricular node or the His‐Purkinje system, and different etiology may result in diverse outcomes. According to the extent of its extension and the characteristics of electrocardiography (ECG), AVB can be categorized into three types: first‐degree AVB, second‐degree AVB, and third‐degree AVB. I AVB is often characterized by excessive prolongation of the PR interval in the electrocardiogram. The PR interval is determined by the conduction time from the sinus node to the ventricles and thus integrates information about a number of sites in the conduction system of the heart. Prolongation of the electrocardiographic PR interval, conventionally known as first‐degree AVB when the PR interval exceeds 200 milliseconds, is frequently encountered in clinical practice.^7^, ^8^, ^9^, ^10^ First‐degree AVB may result from conduction delay in the atrium, atrioventricular node, and/or His‐Purkinje system. The atrioventricular node is the most commonly involved site in adults.^11^Abnormalities in PR interval duration are associated with cardiac conduction defects and increased risk of AF, which carries a substantial risk of morbidity and mortality. However, the reported results of studies on the relationship between I AVB and AF are inconsistent. It is a controversial argument whether I AVB is a pathological state and related to AF. Some studies suggest that individuals with first‐degree AVB are at a substantially increased risk of future AF (~2‐fold) compared to individuals without first‐degree AVB. PR prolongation could be a marker of other changes in the cardiovascular system that contributes to a worse prognosis. Prior studies have raised the possibility that slowed intra‐atrial or interatrial conduction may directly increase the risk of AF.^12^, ^13^, ^14^, ^15^, ^16^, ^17^ An association between PR interval and AF risk has been reported in a separate study that uses Framingham data to construct a clinical risk score for AF,^18^ while others claim that first‐degree AVB has a benign prognosis.^7^, ^9^, ^10^, ^19^, ^20^ First‐degree AVB typically occurs in the absence of acute cardiovascular disease.^7^, ^10^ The relationship between AVB and AF has always been controversial.

Second‐degree AVB is first described in 1899. Second‐degree AVB remains poorly understood despite major advances in cardiac electrophysiology in the past three decades.^21^, ^22^, ^23^, ^24^, ^25^ Because second‐degree AVB can be present in normal children, young adults, and athletes,^26^, ^27^ it does not usually present with any symptoms. However, this is not always the case. The second‐degree AVB is also linked to underlying heart diseases such as intrinsic atrioventricular nodal disease, structural cardiomyopathy, myocarditis, endocarditis, acute inferior myocardial infarction, post cardiac surgery, ablation, catheterization procedures, and secondary to hypothyroidism or hyperthyroidism.^28^ AF associated with AVB is more likely to induce thrombosis and thromboembolic events as well as heart failure and myocardial ischemia due to a slow ventricular rate and electrical remodeling.^29^Whether AF accompanied with second‐degree AVB has clinical significance in the diagnosis of the patient's condition, drug intervention, and treatment guidance, which is worthy of further investigation.

At present, there are not too much controversies about third‐degree AVB. Third‐degree AVB is generally accompanied by the pathological state of the disease and is closely related to the occurrence of the disease. Therefore, the current controversy over third‐degree AVB is not too much exalted. The purpose of this study is to analyze the occurrence of atrioventricular conduction block in patients with AF and non‐AF, and to explore the relationship between the AF and the three types of atrioventricular conduction block, and find out which one is the most common type of AVB occurred in AF patients.

## 2 METHODS

**Figure 1:**
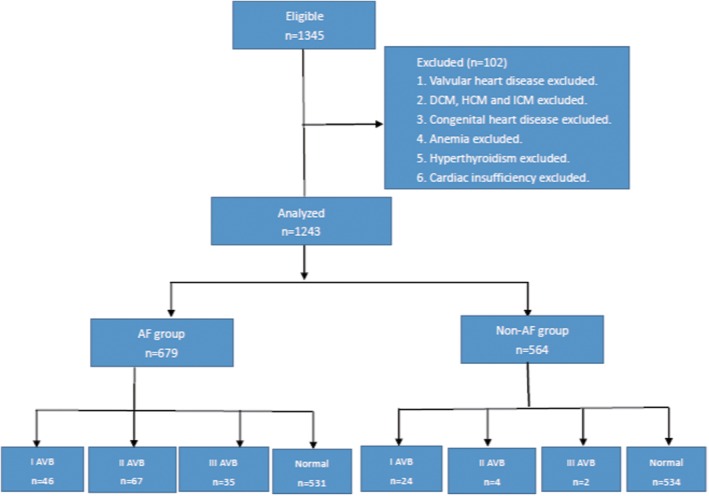
The above flow chart shows the design of inclusion and exclusion of atrial fibrillation (AF) and non‐AF group. After excluding 102 patients, 679 patients with atrial fibrillation and 564 patients without atrial fibrillation were finally included in the Atrial Fibrillation center of Xi 'an Jiaotong University. To study the relationship between AVB and atrial fibrillation, I AVB,II AVB and III AVB in the atrial fibrillation group and the non‐atrial fibrillation group were respectively compared

1. The clinical data process of AF and non‐AF groups (Figure 1):
  Figure 1 shows the whole process of the data collection and analysis.
2. The inclusion and exclusion criteria of AF and non‐AF:
  The inclusion criteria of AF:
  1. More than two episodes of AF occurred and recorded by ECG before.
  2. Aged from 18 to 80 years old.
  The exclusion criteria of AF:
  1. Valvular heart disease and rheumatic heart disease excluded.
  2. Dilated cardiomyopathy, hypertrophic cardiomyopathy, and ischemic cardiomyopathy excluded.
  3. Congenital heart disease excluded.
  4. Anemia excluded.
  5. Hyperthyroidism excluded.
  6. Cardiac insufficiency (EF < 35%) excluded.
3. Diagnosis of AVB
  I AVB:
  The PR interval is greater than 0.2 seconds, and each atrial excitement is transmitted to the ventricle.
  II AVB of Type I:
  ECG shows a gradual extension of time from the atrium to the ventricle, and an atrial excitement cannot be transmitted to the ventricle.
  II AVB of Type II:
  It means that the atrial impulse cannot be transmitted to the ventricle, and the ECG is represented as the leakage of QRS interphase.
  III AVB:
  It means that all atrial excitations cannot be transmitted to ventricles, and the activities of atria and ventricles are independent, respectively, and unrelated to each other.
4. Case selection

This experiment is designed to study the relationship between AVB and AF. The study retrospectively reviewed 1345 consecutive patients from August 2017 to August 2018, who were diagnosed with AF and non‐AF. All patients were enrolled from the Atrial Fibrillation Center, Department of Cardiology, the First Affiliated Hospital of Xi'an Jiaotong university. The clinical data were obtained from medical record review and analyzed in AF center. Patients were divided into the AF group and non‐AF group. Among the total of 1345 patients, 102 patients who did not meet the inclusion criteria were excluded:26 cases were diagnosed with Valvular heart disease; 12 cases had dilated heart disease; 6 cases had hypertrophic heart disease; 4 cases had ischemic cardiomyopathy; 17 cases had Congenital heart disease; 18 cases had anemia; 17 cases had hyperthyroidism; and 4 presented with severe left ventricle dysfunction (EF < 35%). Meanwhile, patients in the AF group must comply with the following conditions: (a) more than two episodes of AF occurred and recorded by ECG before and (b) aged from 18 to 80. The final total sample size was 1243 patients, including 679 patients in the AF group (378, 55.7% males) and 564 patients in the non‐AF control group (287, 50.8% males).

## 3 STATISTICAL ANALYSIS

Independent sample *t* test and Pearson *χ* ^2^ test were used to compare the continuous variables and the classified variables. Single factor analysis method was used to preliminarily analyze related risk factors. Statistically significant factors were taken into multiple factors analysis model by using negative binomial regression for the analysis of the related factors. The aim is to evaluate whether there is statistical significance between AF and corresponding covariates in the negative binomial regression model. Finally, the correlation between AVB interval and AF was found.

## 4 RESULT

### 4.1 The distribution of AVB in AF and non‐AF groups

From Table 1, we found that atrioventricular conduction block of degree I, degree II, and degree III increased in the patients with AF compared with patients with non‐AF.

**Table 1:** The table above shows the proportion and comparison of three types of AVB in the AF group and the non‐AF group

Category	AF	Non‐AF	Odds ratio	*P*‐value
Normal	531 (78.2%)	534 (94.68%)		*P* > 0.05
Abnormal	148 (21.8%)	30 (5.32%)	4.961 (3.291，7.479)	*P* = 0.001
I AVB	46 (6.77%)	24 (4.26%)	1.927 (1.160,3.203)	*P* = 0.013
II AVB	67 (9.87%)	4 (0.71%)	16.845 (6.099，46.524)	*P* = 0.000
III AVB	35 (5.15%)	2 (0.35%)	17.599 (4.212，73.541)	*P* = 0.000

Abbreviations: AF, atrial fibrillation; AVB, atrioventricular block.

The I AVB has a relative statistical risk of 1.927 (95% CI: 1.160‐3.203, *P* < 0.05) with the occurrence of AF. II AVB occupies the largest proportion, accounting for 67 cases (9.87%), and the statistical risk of II AVB of AF is 16.845 (95% CI: 6.099‐46.524, *P* < 0.000) compared with the non‐AF group. III AVB has a relative statistical risk of 17.599 (95% CI: 4.212‐73.541, *P* < 0.000) in the AF group compared with the non‐AF group.

According to the analysis results of this study, second atrioventricular conduction block occupies the largest percentage, accounting for 67 cases (9.87%) among the three types of atrioventricular conduction block in the AF group. The correlation between second atrioventricular conduction block and AF is the most obvious (*P* < 0.000).

### 4.2 Univariate analysis of AF and its related variables are performed

The statistical results are shown in Table 2. We include relevant variables of the AF group and the non‐AF group into the univariate analysis model, and conduct statistical analysis between different categories of continuous variables and classified variables, respectively. The gender, age, body mass index, systolic blood pressure (SBP), diastolic blood pressure (DBP), heart rate, AVB, hypertension, diabetes, kidney disease, coronary heart disease, cerebral infarction, red blood cell (RBC), white blood cell, platelet count (PLT), aspartate aminotransferase (AST), alanine transaminase (ALT), total cholesterol (CHOL), triglycerides (TG), low‐density lipoprotein (LDL), high‐density lipoprotein, urea (BUN), creatinine (CRE), creatine kinase (CK), creatine kinase isozyme (CKMB), Prothrombin standardized ratio (INR), Fibrinogen degradation products (FDP), Hba1c (HGB), potassium (K), sodium (NA), chlorine (CL), FT4 free thyroxine (FT4), FT3 free thyroxine (FT3), h‐tsh thyrotropin (TSH), QRS interval (QRS), left atrial diameter, left ventricular diameter, ejection fraction (EF), and cardiac output (CO) in both the AF group and the non‐AF group were analyzed by univariate analysis, respectively, and the following results have statistical significance: age (95% CI: 1.047‐1.068, *P* < 0.000), SBP (95% CI: 0.967‐0.978, *P* < 0.000), DBP (95% CI: 0.963‐0.980, *P* < 0.000), RBC (95% CI: 0.533‐0.815, *P* < 0.000), PLT (95% CI: 0.988‐0.993, *P* < 0.000), CHOL (95% CI: 0.895‐0.963, *P* < 0.000), TG (95% CI: 0.627‐0.817, *P* < 0.000), FDP (95% CI: 1.103‐1.323, *P* < 0.000), HGB (95% CI: 1.178‐1.627, *P* < 0.000), K (95% CI: 1.649‐2.911, *P* < 0.000), QRS (95% CI: 0.000‐0.030, *P* < 0.05), left atrial diameter (95% CI: 1.281‐1.372, *P* < 0.000), left ventricular diameter (95% CI: 1.006‐1.057, *P* < 0.05), EF (95% CI: 0.911‐0.946, *P* < 0.000), AVB (95% CI: 3.291‐7.479, *P* < 0.000), CO (95% CI: 1.072‐1.263, *P* < 0.000), hypertension (95% CI: 0.198‐0.332, *P* < 0.01), coronary heart disease (95% CI: 1.768‐3.431, *P* < 0.01), and cerebral infarction (95% CI: 2.601‐11.773, *P* < 0.01). The above statistical factors were then included in the multivariate analysis model for further correlation analysis.

**Table 2:** The above table shows the results of univariate analysis of related factors in the AF and non‐AF groups

Category	AF	Non‐AF	Odds ratio (95% confidence interval)	*P*
Gender	Male (378，55.7%) Female (301，44.3%)	Male (287，50.8%) Female (278，49.2%)	1.212 (0.969‐1.516)	*P* = 0.092
Age (*r*)	66.57 ± 11.923	57.85 ± 13.033	1.058 (1.047‐1.068)	*P* < 0.000***^a^
Body mass index	25.08 ± 4.909	25.12 ± 3.355	1.000 (0.993‐1.027)	*P* = 0.999
Heart rates	77.64 ± 19.125	77.36 ± 12.818	1.001 (0.994‐0.008)	*P* = 0.773
Systolic blood pressure (mm Hg)	125.59 ± 18.571	138.08 ± 24.487	0.973 (0.967‐0.978)	*P* < 0.0001***
Diastolic blood pressure (mm Hg)	76.91 ± 14.581	83.01 ± 15.593	0.972 (0.963‐0.980)	*P* < 0.0001***
Red blood cell	4.46 ± 0.55	4.58 ± 0.53	0.659 (0.533‐0.815)	*P* = 0.000***
White blood cell	6.05 ± 1.79	6.26 ± 1.84	0.940 (0.884‐1.000)	*P* = 0.051
PLT	177.93 ± 56.32	209.59 ± 58.65	0.990 (0.988‐0.993)	*P* = 0.000***
AST	23.97 ± 14.5	23.54 ± 23.91	1.001 (0.995‐1.007)	*P* = 0.706
ALT	25.97 ± 25.75	26.95 ± 32.87	0.999 (0.995‐1.003)	*P* = 0.563
CHOL	4.33 ± 3.52	5.34 ± 4.50	0.928 (0.895‐0.963)	*P* = 0.000***
TG	1.31 ± 0.92	1.74 ± 2.64	0.715 (0.627–0.817)	*P* = 0.000***
Low‐density lipoprotein	2.84 ± 20.27	2.30 ± 0.79	1.003 (0.992‐1.004)	*P* = 0.588
High‐density lipoprotein	1.05 ± 0.38	1.33 ± 6.13	0.956 (0.765‐1.194)	*P* = 0.690
BUN	6.34 ± 2.91	5.58 ± 1.88	1.192 (1.118‐1.271)	*P* = 0.000***
CRE	69.1 ± 23.66	65.09 ± 27.22	1.007 (1.002‐1.013)	*P* = 0.008**
CK	86.57 ± 54.02	96.04 ± 80.44	0.998 (0.996‐1.000)	*P* = 0.020*
CKMB	13.31 ± 8.65	12.84 ± 15.50	1.003 (0.993‐1.013)	*P* = 0.520
INR	1.43 ± 2.94	1.19 ± 3.97	1.030 (0.975‐1.088)	*P* = 0.288
FDP	2.61 ± 9.51	1.42 ± 1.12	1.208 (1.103‐1.323)	*P* = 0.000***
HGB	5.92 ± 0.88	5.72 ± 0.75	1.384 (1.178‐1.627)	*P* = 0.000***
K	4.63 ± 15.63	3.88 ± 0.41	2.191 (1.649‐2.911)	*P* = 0.000***
NA	142.3 ± 40.23	142.80 ± 4.63	0.999 (0.996‐1.003)	*P* = 0.775
CL	100.67 ± 10.16	100.26 ± 6.70	1.005 (0.992‐1.019)	*P* = 0.421
FT4	14.74 ± 3.97	14.71 ± 3.16	1.002 (0.969‐1.035)	*P* = 0.918
FT3	4.84 ± 7.04	4.78 ± 0.99	1.002 (0.979‐1.026)	*P* = 0.856
TSH	3.13 ± 5.18	2.75 ± 3.20	1.024 (0.989‐1.060)	*P* = 0.183
QRS	0.097 ± 0.020	0.1 ± 0.013	0.000 (0.000‐0.030)	*P* = 0.003**
Left atrial diameter	39.14 ± 6.59	31.75 ± 3.90	1.326 (1.281‐1.372)	*P* = 0.000***
Left ventricular diameter	46.37 ± 5.83	45.56 ± 4.4	1.031 (1.006‐1.057)	*P* = 0.016*
EF	64.37 ± 8.25	67.99 ± 5.94	0.928 (0.911‐0.946)	*P* = 0.000***
CO	5.97 ± 1.69	5.61 ± 1.36	1.164 (1.072‐1.263)	*P* = 0.000***
AVB	Yes 148 (21.80%) No 531 (78.20%)	Yes 30 (5.32%) No 534 (94.68%)	4.961 (3.291‐7.479)	*P* = 0.000***
Cerebral infarction	Yes 50 (7.4) No 628 (92.5)	Yes 8 (1.4) No 556 (98.6)	5.533 (2.601‐11.773)	*P* = 0.000***
Hypertension	Yes 351 (51.7) No 328 (48.3)	Yes 455 (80.7) No 109 (19.3)	0.256 (0.198‐0.332)	*P* = 0.000***
Diabetes	Yes 120 (17.7) No 559 (82.3)	Yes 80 (14.2) No 484 (85.8)	1.299 (0.955‐1.767)	*P* = 0.096
Coronary heart disease	Yes 145 (21.4) No 533 (78.5)	Yes 56 (9.9) No 508 (90.1)	2.463 (1.768‐3.431)	*P* = 0.000***
Chronic kidney disease	Yes 9 (1.3) No 670 (98.7)	Yes 13 (2.3) No 551 (97.7)	0.569 (0.242‐1.342)	*P* = 0.191

Abbreviations: AF, atrial fibrillation; AVB, atrioventricular block.

*P*‐value from *t* tests for continuous variables and *χ* ^2^ tests for categorical variables. *P* value less than 0.05 is indicated by *; *P* value less than 0.01 is marked with **; *P* value less than 0.001 is marked with ***

### 4.3 Negative binomial regression analysis of AF and non‐AF groups

We select variables to be included in the multivariate regression model by consulting relevant literature. Variables to be included are selected by DAG diagram and results of univariate analysis. As it is shown in Table 3, we incorporate the following factors into the multivariate regression model: age, gender, AVB, left atrial diameter, hypertension, coronary heart disease, diabetes, and cerebral infarction. The above factors are analyzed by negative binomial regression for further correlation analysis. The following results have statistical significance: The no AVB/AVB (95% CI: 0.811 0.722‐0.911, *P* < 0.000), age (95% CI: 1.011 1.006‐1.016, *P* < 0.000), left ventricular diameter (95% CI: 1.060 1.049‐1.071, *P* < 0.000), cerebral infarction (95% CI: 1.311 1.106‐1.554, *P* < 0.01), hypertension (95% CI: 0.588 0.531‐0.652, *P* < 0.000), coronary heart diseases (95% CI: 1.201 1.061‐1.360, *P* < 0.01), diabetes (95% CI: 1.126 0.987‐1.285, *P* > 0.05), and gender (95% CI: 1.059 0.952‐1.178, *P* > 0.05). We set AF as a dependent variable, related factors are included into the multifactor analysis model. Finally, after adjusting age, gender, left atrial diameter, hypertension, diabetes, coronary heart diseases, and cerebral infarction, the no AVB/AVB still have a relative statistical risk of 0.811 (95% CI: 0.722‐0.911, *P* < 0.000) with the occurrence of AF.

**Table 3:** Negative binomial regression results between AF and non‐AF

Category	Univariate analysis	Negative binomial regression	95% confidence interval for Exp (B)
No AVB/AVB	*P* = 0.000***	*P* = 0.000***	0.811 (0.722‐0.911)
Age (*r*)	*P* = 0.000***	*P* = 0.000***	1.011 (1.006‐1.016)
Gender (male)	*P* = 0.092	*P* = 0.292	1.059 (0.952–1.178)
Left atrial diameter	*P* = 0.000***	*P* = 0.000***	1.060 (1.049‐1.071)
Cerebral infarction	*P* = 0.000***	*P* = 0.002**	1.311 (1.106‐1.554)
Hypertension	*P* = 0.000***	*P* = 0.000***	0.588 (0.531‐0.652)
Coronary heart disease	*P* = 0.000***	*P* = 0.004**	1.201 (1.061‐1.360)
Diabetes	*P* = 0.096	*P* = 0.078	1.126 (0.987‐1.285)

Abbreviations: AF, atrial fibrillation; AVB, atrioventricular block. *P* value less than 0.01 is marked with **; *P* value less than 0.001 is marked with ***

## 5 DISCUSSION

AF is the most common sustained cardiac arrhythmia in clinical practice, and its incidence is increasing rapidly worldwide accounting for nearly one‐third of all hospital admissions.^1^ It is also associated with increased risks of stroke, heart failure, cognitive dysfunction, impaired quality of life, and substantial healthcare costs. Furthermore, it eventually contributes to increased risks of thromboembolic events,^2^ cardiac, and overall mortalities,^3^, ^4^, ^5^, ^6^ which may result in different outcomes. AVB is categorized into three types according to the extent of its extension and the characteristics of ECG: first‐degree AVB, second‐degree AVB, and third‐degree AVB.

### 5.1 I AVB and AF

The relationship between I AVB and arrhythmia has been controversial. The reported results of studies on the relationship between I AVB and AF are inconsistent. Some studies suggest that individuals with first‐degree AVB are at a substantially increased risk of future AF (~2‐fold)^8^ compared with individuals without first‐degree AVB. An association between PR interval and AF risk has been reported.^18^PR prolongation could be a marker of worse prognosis in the cardiovascular system. Potential explanations may account for the association of longer PR interval with AF risk. In addition, a prolonged PR interval results in delayed and ineffective mitral valve closure and diastolic mitral regurgitation,^30^ especially when the PR interval exceeds 230 milliseconds,^31^ while others claim that first‐degree AVB has a benign prognosis,^7^, ^9^, ^10^, ^19^, ^20^ although these studies are based on young, healthy men in the military.^7^, ^19^ First‐degree AVB typically occurs in the absence of acute cardiovascular disease.^7^, ^10^ The relationship between I AVB and AF has always been controversial.

Through the statistical analysis of the AF data in the Atrial Fibrillation Center of The First Affiliated Hospital of Xi'an Jiaotong University during 2017‐2018, it is found that: I AVB is increased in the AF group (n = 46, 6.77%) compared with the non‐AF group (24, 4.26%). The I AVB has a relative statistical risk of 1.927 (95% CI: 1.160‐3.203, *P* < 0.05) with the occurrence of AF. I AVB is a risk factor for AF.

### 5.2 II AVB and AF

Second‐degree AVB is often associated with an underlying heart disease such as atrioventricular node disease, structural cardiomyopathy, myocarditis, endocarditis, acute inferior myocardial infarction, post cardiac surgery, ablation, catheterization procedures, and secondary to hypothyroidism or hyperthyroidism.^28^ Furthermore, AF associated with AVB is more likely to induce thrombosis and thromboembolic events as well as heart failure and myocardial ischemia due to a slow ventricular rate and electrical remodeling.^29^ Sometimes II AVB does not usually present with any symptoms, but it can be present in normal children, young adults, and athletes.^26^, ^27^ Whether AF accompanied with second‐degree AVB has clinical significance for the diagnosis of the patient's condition, drug intervention, and guidance is worth being further explored.

The relationship between II AVB and arrhythmia is not as controversial as I AVB is. By data analysis, among the three types of AVB in the AF group, II AVB occupied the largest proportion, accounting for 67 cases (9.87%); II AVB holds the statistical risk of 16.845 (95% CI: 6.099‐46.524, *P* < 0.000) compared with the non‐AF group. II AVB is an obvious risk factor for AF.

### 5.3 III AVB and AF

At present, there are not too much reports and controversies over III AVB and AF. III AVB is usually accompanied with the pathological state of the disease and is closely related to the occurrence of the disease. Therefore, the current controversy over III AVB is not too much great. Through the statistical analysis of the data, we draw the conclusion that: III AVB has a relative statistical risk of 17.599 (95% CI: 4.212‐73.541, *P* < 0.000) in the AF group compared with the non‐AF group. III AVB is also an obvious risk factor for AF.

To sum up, the three types of AVB are risk factors for AF. The proportion of three types of AVB in the AF group is, respectively, increased compared with the non‐AF group, and all of them have statistical significance. The I AVB has a relative statistical risk of 1.927 (95% CI: 1.160‐3.203, *P* < 0.05) with the occurrence of AF. II AVB takes the largest percentage, accounting for 67 cases (9.87%), and the statistical risk of II AVB in AF is 16.845 (95% CI: 6.099‐46.524, *P* < 0.000) compared with the non‐AF group. III AVB has a relative statistical risk of 17.599 (95% CI: 4.212‐73.541, *P* < 0.000) in the AF group compared with the non‐AF group. Although I AVB has been controversial, this study agrees that I AVB is a risk factor for AF. The comparatively relevant and highest incidence of AVB is II AVB. Second‐degree AVB is the most correlated type with AF among the three types of AVB. At the same time, third‐degree atrioventricular conduction block is also strongly related to the occurrence of AF. The results point out that II and III degree AVB are also risk factors for AF.

The three types of AVB are risk factors for AF occurrence, and II AVB has the highest proportion and high correlation with the occurrence of AF.

### 5.4 Limitation

This study is an observational research conducted in a single center. The research center is relatively single, and the sample representation is not enough.

## CONFLICT OF INTEREST

The authors declare no potential conflict of interests.

## Supporting information

Figure S1
