# Supplementary figures and images for: Atrioventricular block can be used as a risk predictor of clinical atrial fibrillation
Source: Clin Cardiol. 2019 Mar 18;42(4):452–8. doi: 10.1002/clc.23167 (PMC6712334; doi:10.1002/clc.23167)

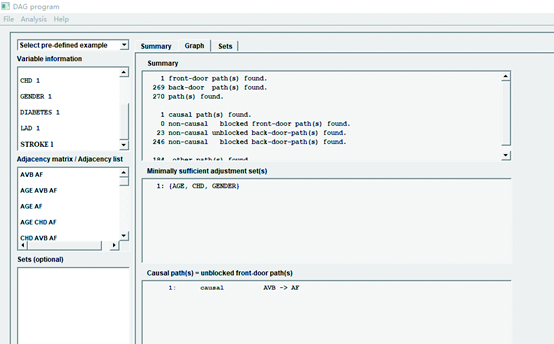

Supplement: Supplementary file 1 — Figure S1 [file CLC-42-452-s001.tif]
